# Real-Time Detection and Localization of Force on a Capacitive Elastomeric Sensor Array Using Image Processing and Machine Learning

**DOI:** 10.3390/s25103011

**Published:** 2025-05-10

**Authors:** Peter Werner Egger, Gidugu Lakshmi Srinivas, Mathias Brandstötter

**Affiliations:** ADMiRE Research Center, Carinthia University of Applied Sciences, 9524 Villach, Austria; peter.egger@alumni.fh-kaernten.at (P.W.E.); m.brandstoetter@fh-kaernten.at (M.B.)

**Keywords:** soft tactile sensors, image processing techniques, force localization, machine learning models, soft robotics

## Abstract

Soft and flexible capacitive tactile sensors are vital in prosthetics, wearable health monitoring, and soft robotics applications. However, achieving accurate real-time force detection and spatial localization remains a significant challenge, especially in dynamic, non-rigid environments like prosthetic liners. This study presents a real-time force point detection and tracking system using a custom-fabricated soft elastomeric capacitive sensor array in conjunction with image processing and machine learning techniques. The system integrates Otsu’s thresholding, Connected Component Labeling, and a tailored cluster-tracking algorithm for anomaly detection, enabling real-time localization within 1 ms. A 6×6 Dragon Skin-based sensor array was fabricated, embedded with copper yarn electrodes, and evaluated using a UR3e robotic arm and a Schunk force-torque sensor to generate controlled stimuli. The fabricated tactile sensor measures the applied force from 1 to 3 N. Sensor output was captured via a MUCA breakout board and Arduino Nano 33 IoT, transmitting the Ratio of Mutual Capacitance data for further analysis. A Python-based processing pipeline filters and visualizes the data with real-time clustering and adaptive thresholding. Machine learning models such as linear regression, Support Vector Machine, decision tree, and Gaussian Process Regression were evaluated to correlate force with capacitance values. Decision Tree Regression achieved the highest performance ($R^{2}=0.9996,\;RMSE=0.0446$), providing an effective correlation factor of 51.76 for force estimation. The system offers robust performance in complex interactions and a scalable solution for soft robotics and prosthetic force mapping, supporting health monitoring, safe automation, and medical diagnostics.

## 1. Introduction

### 1.1. Motivation

Limb amputation significantly impacts an individual’s mobility, independence, and overall quality of life. In 2019, approximately 13.23 million new cases of traumatic amputations were reported globally, marking a 16.4% increase since 1990 [1]. The prevalence of individuals living with amputations rose from 370.25 million in 1990 to 552.45 million in 2019, a 49.2% increase over nearly three decades. Diabetes remains a major contributor to limb loss, with 537 million adults (20 to 79 years) diagnosed globally in 2021. This number is projected to rise to 783 million by 2045, a 46% increase [2]. Prosthetic devices play a vital role in restoring mobility; however, up to 57% of users abandon their prostheses due to poor fit, discomfort, and skin irritation [3,4]. The interface between the residual limb and the prosthesis affects stability, load distribution, and overall comfort [5]. Achieving an optimal fit remains challenging due to the dynamic nature of residual limb volume, pressure distribution inconsistencies, and mechanical friction, often leading to pressure sores, pain, and reduced device usability [6].

To address these challenges, sensorized prosthetic liners have been developed to monitor pressure distribution and improve comfort [7]. Traditional prosthetic pressure sensors often use piezoresistive, piezoelectric, or capacitive technologies, each with advantages and limitations [8]. Capacitive sensors, in particular, offer high sensitivity, low power consumption, and flexibility, making them ideal for wearable applications and soft robotics [9]. However, a major challenge in these sensors is real-time data interpretation and adaptive response to external stimuli. Most existing sensor-based prosthetic systems rely on rigid sensor arrays, which lack adaptability to the changing shape of the residual limb [10]. Furthermore, accurate force localization and clustering of pressure points remain largely unexplored. While previous studies have integrated capacitive sensors in prosthetic applications, they primarily focus on raw data collection rather than intelligent data interpretation. Machine learning and image processing techniques offer promising real-time force detection, clustering, and spatial pressure mapping solutions [11]. By applying unsupervised learning methods, such as clustering algorithms, connected-component labeling, and adaptive filtering, capacitive sensor data can be processed efficiently to identify meaningful pressure distributions and automate pressure point detection [12]. Image processing algorithms further enhance localization accuracy, enabling real-time visualization of sensor responses and adaptive force estimation. This study aims to integrate a soft capacitive sensor array with machine learning-based clustering and image processing techniques to enhance force characterization and localization. The proposed system achieves real-time detection and adaptive response by leveraging unsupervised learning and image segmentation techniques, making it highly applicable for prosthetic liners and robotic touch sensing. This approach improves prosthetic comfort and usability and provides a scalable real-time tactile force monitoring solution in soft robotics and assistive technologies.

### 1.2. Related Work

Several researchers have focused on developing soft capacitive sensors for various force-sensing applications. Zini et al. developed a flexible capacitive sensor for wearable applications, demonstrating its effectiveness in continuous pressure monitoring [13]. The advancement of artificial skin sensors focuses on improving human–robot interactions by mimicking the tactile feedback essential for human communication. Initial developments, including force-sensing resistors, provided a cost-effective solution but encountered difficulties due to intricate wiring and multilayered designs [14,15]. Qu et al. introduced an elastomer-based capacitive sensor array for soft robotic skins, achieving high force sensitivity and flexibility [16]. Similarly, a biocompatible capacitive sensor was developed for prosthetic applications to enable real-time force mapping [17]. However, these studies primarily focused on sensor design and fabrication techniques, with limited emphasis on real-time data processing and clustering algorithms. Capacitive sensors have been integrated into prosthetic liners for pressure monitoring, offering enhanced fit assessment and user comfort. A sensorized liner system utilizing quantum technology-based pressure sensors has been developed, though it requires manual calibration before each use [18]. Another approach introduced a low-cost, flexible capacitive sensor for prosthetic fit monitoring, but it lacked automated clustering and adaptive processing capabilities [19]. These developments demonstrate the potential of capacitive sensors in medical applications but also highlight the necessity for intelligent data processing algorithms to improve sensor efficiency, adaptability, and usability.

Machine learning techniques have been increasingly adopted for sensor characterization, clustering, and force estimation, significantly enhancing capacitive sensor applications. Deep neural networks have been applied to estimate forces with high precision, although they require large training datasets [20]. A Support Vector Machines (SVM)-based model has been utilized to classify sensor responses, improving pattern recognition, yet lacking real-time adaptability [21]. Further studies have employed unsupervised clustering methods to distinguish multiple contact points on capacitive touch surfaces, demonstrating the feasibility of automated data segmentation [22]. Machine learning enhances tactile sensor accuracy and response times by correlating sensor outputs with external forces, benefiting real-time applications in robotics and soft robotics [23]. Models like linear regression, decision trees, and random forests effectively predict force and pressure from capacitive data, enabling more adaptive robotic systems [24]. While these techniques show promise, their integration with image processing for real-time visualization and adaptive clustering remains an open challenge.

Image processing techniques are key in visualizing contact forces and mapping pressure distribution. Otsu’s widely used thresholding algorithm method has been applied to tactile sensors for background noise removal and signal enhancement [25]. A real-time Connected Component Labeling method has been developed for contact force segmentation, demonstrating its effectiveness in multi-contact interaction scenarios [26]. Despite advancements in clustering and segmentation, research integrating image processing with clustering algorithms for real-time force distribution analysis remains limited. This study aims to bridge this gap by combining Otsu’s thresholding, Connected Component Labeling, and clustering methods to achieve real-time sensor data interpretation and pressure point tracking, ensuring greater accuracy and adaptability in capacitive sensing applications.

### 1.3. Contribution and System Overview

This work presents a machine learning-enhanced capacitive sensor system that integrates real-time clustering and image processing techniques for force sensing and localization. A soft elastomeric sensor detects pressure points, while machine learning algorithms analyze sensor force characterization based on capacitance. Otsu’s thresholding and Connected Component Labeling enhance pressure point localization, ensuring precise segmentation and tracking. The system undergoes experimental validation using a UR3e robotic arm, which applies controlled forces to evaluate sensor response and accuracy. The system, designed for prosthetic liners and soft robotics, optimizes force distribution analysis, enhances sensor adaptability, and enables real-time visualization to improve user comfort.

**Figure 1:**
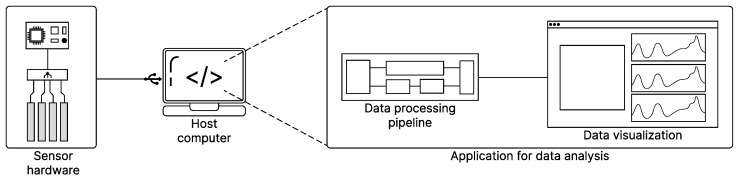
Overview of the components used and developed in this work.

Figure 1 presents an overview of the system; it consists of a soft capacitive sensor, a hardware interface, and a software application for data processing and visualization. A microcontroller board reads the sensor data and transmits them via USB to the host system, where a dedicated hardware interface ensures proper connectivity. The software application processes incoming data, applies clustering and image processing techniques, and visualizes pressure points in real time, enabling efficient force localization. This integrated system architecture allows for precise pressure mapping and adaptive analysis, making it suitable for prosthetic and robotic applications.

## 2. Methodology

### 2.1. Capacitive Sensing Principle

**Figure 2:**
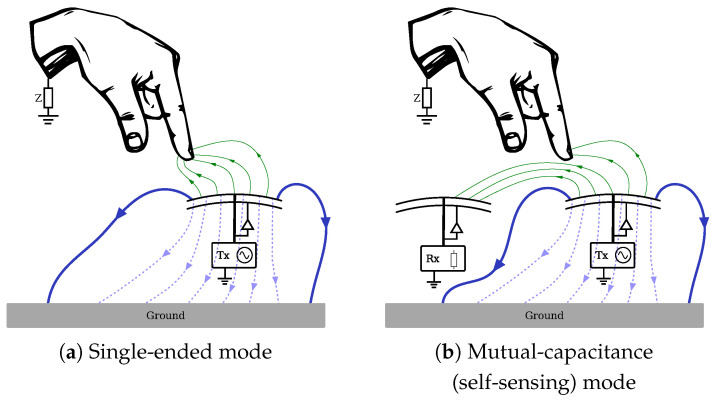
Illustration of the capacitive sensing principles. In (**a**), electrical potential is between the electrode and the hand; in (**b**), an electrical field is between Rx and Tx, but is partially intercepted by a conductive object.

The capacitive sensor used in this work operates in the mutual capacitance mode, a commonly applied principle in multi-touch and force-sensitive systems, as shown in Figure 2b. When an external force is applied to the soft elastomeric structure, the material deforms, causing changes in the distance between the electrodes and the overlapping area. These mechanical alterations lead to a variation in the measured capacitance. The deformation behavior follows the Poisson effect, where lateral contraction occurs when a material is compressed longitudinally. On the other hand, in single-ended configuration shown in Figure 2a, the capacitance is measured between each sensing electrode and a fixed reference electrode, typically connected to the ground, enabling localized detection of pressure-induced changes.

The capacitive sensor consists of two interlocking layers of conductive wires with a 90° rotation with a synthetic layer between them. Connector pins were soldered to the wires’ endings to drive the sensor and read its data. Embedding this structure in soft silicone protects the circuitry against wear and fluids. Local capacitance cells are formed when the connector pins supply the sensor with electricity. The value of a capacity *C* is expressed in Farad and depends on the area *A* of the electrodes and the distance *d* between the electrodes, as follows:

(1) $$C=\epsilon_{0}\;\epsilon_{r}\frac{A}{d},$$

where $\epsilon_{r}$ represents the dielectric constant, and $\epsilon_{0}$ denotes the vacuum permittivity. Mutual capacitance technology enables multi-point detection, improved accuracy, and finger-hover sensing before contact. Electrode geometry is crucial in sensitivity, with different patterns affecting resolution. While diamond-shaped electrode patterns improve efficiency, this study employs a rectangular electrode layout, balancing simplicity in fabrication with reliable performance. Electrode spacing and thickness further influence the system’s resolution and capacitance measurement accuracy, optimizing it for real-time force sensing applications.

### 2.2. Elastomeric Sensor Array

**Figure 3:**
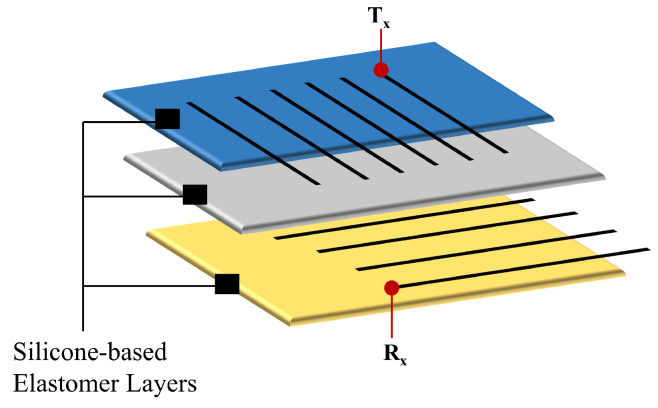
Prototype of the soft elastomeric sensor array.

A 6×6 matrix sensor patch was developed using silicone due to its strain resistance and viscoelastic properties, making it highly suitable for orthotics and soft robotics applications. A 4 mm-thick Dragon Skin silicone layer with a Shore hardness of 2 A was incorporated as the outermost covering to insulate the sensing electrodes and prevent direct contact. This material was chosen for its soft, skin-like texture, enhancing comfort in wearable applications [27]. The sensor’s electrode grid was arranged with uniform spacing on this insulating layer, ensuring consistent performance, as depicted in Figure 3. To secure and shield the electrodes while maintaining flexibility, a 5 mm Ecoflex layer with a Shore hardness of 30 was applied, reinforcing the structure under mechanical strain [28]. The sensing layer consists of conductive yarn electrodes designated as Tx and Rx. These electrodes, made from 0.2 mm-thick copper yarn with a resistance per unit length of 4.2 Ω/m, ensure a compact and flexible design while preserving the silicone’s mechanical properties.

The sensor’s layered configuration allows it to conform to curved surfaces while maintaining robustness and energy absorption. Unlike conventional shielding layers, the topmost layer in this design mirrors the properties of the bottom layer, providing structural integrity and consistency across the entire patch. The electrodes were soldered onto the sensing board, ensuring durable and flexible connection optimized for real-world applications.

### 2.3. Data Acquisition and Characterization System

**Figure 4:**
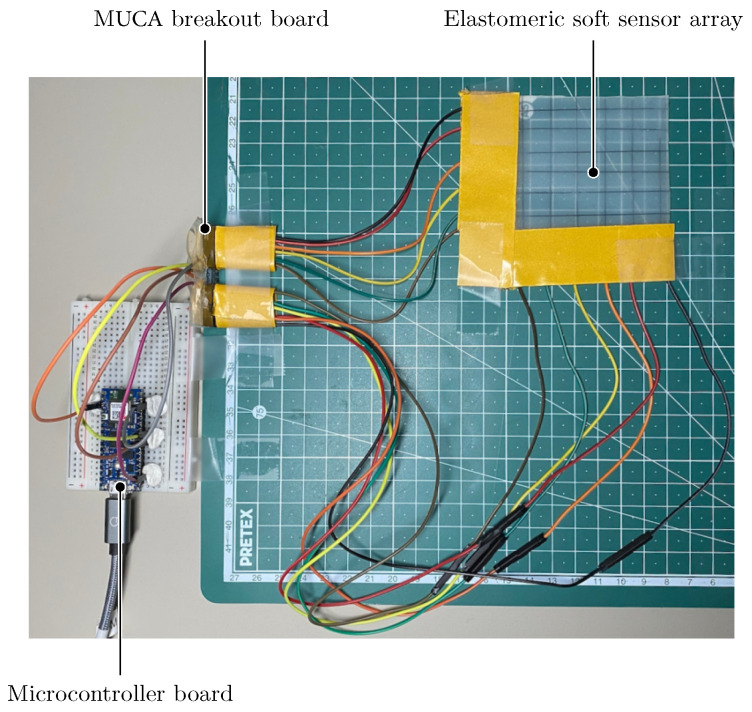
Hardware setup of the Data Acquisition System.

The Data Acquisition System (DAQ) captures real-world phenomena, converts them into digital signals, and transmits the data to a host computer for further processing and analysis. In this work, the acquisition system consists of a soft capacitive sensor connected to a hardware interface and a microcontroller, which handles data transmission to the computer. The MUCA breakout board (re-facto, Odoo S.A., Ramillies, Walloon Brabant, Belgium) was selected for this purpose. It is an open-source mutual capacitance board designed for multi-touch sensing and integrates the FT5316DME capacitive touch panel controller from FocalTech (FocalTech Systems Co., Ltd., Zhubei City, Hsinchu, 302047, Taiwan). The board supports 21 transmit and 12 receive electrodes, with electrode driving capability up to 15 kΩ, and can detect mutual capacitance values from 1 pF to 4 pF. Communication with the processor occurs via an Inter-Integrated Circuit (I^2^C) interface, with a low power consumption of approximately 0.05 mAh per scan [29,30]. The MUCA board supports simultaneous tracking of up to five contact points and provides raw sensor data at 16-bit resolution across all intersections of the electrode grid. The MUCA board was interfaced with an Arduino Nano 33 IoT microcontroller (Somerville, MA, 02116, USA) via its I^2^C-enabled ports, as illustrated in Figure 4. The microcontroller continuously reads sensor values and transmits them via USB to a host computer for processing. The sensor’s transmit (Tx) and receive (Rx) lines were connected to the MUCA board’s corresponding pins, enabling accurate mapping of electrode interactions during deformation.

To evaluate and characterize the sensor’s performance, controlled force inputs were applied using a UR3e collaborative robotic arm (Universal Robots A/S, Odense, 5000, Denmark). The robot offers a repetition accuracy of ±0.1 mm and was programmed via its teach pendant to deliver precise vertical displacements to selected sensor locations. A single-point probe, fabricated using a Prusa 3D printer (Prusa Research a.s., Prague 7, 17000, Czech Republic) with PLA, was attached to the robot’s end effector. A SCHUNK AXIA80 force/torque sensor (SCHUNK GmbH, Lauffen am Neckar, 74348, Germany) was integrated between the probe and the robotic arm to measure the applied force during testing. The firmware of the Arduino was programmed in C++ with Visual Studio Code, and the PlatformIO plugin [31,32]. The Arduino Nano 33 IoT firmware was written in C++ using Visual Studio Code and the PlatformIO plugin. It was adapted from the publicly available MUCA example sketch to suit the specific dimensions of the fabricated soft sensor [33].

The poetry package manager [34] was used to manage Python 3.11 dependencies; the most important ones were matplotlib@3.7.1, numpy@1.24.3, pandas@1.5.3, scikit-learn@1.2.2, and pyserial@3.5. In the main loop, the firmware reads values from each sensor cell. It transmits them over the serial interface as a comma-separated string of decimal values, as defined in Equation (2). The setup enables real-time sensor data monitoring by capturing and analyzing variations in electrical charge distribution across the sensor array.

(2) $$\left[\begin{array}{cccc} x_{1,1} & x_{1,2} & \ldots & x_{1,m}\\ x_{2,1} & x_{2,2} & \ldots & x_{2,m}\\ \vdots & \vdots & \ddots & \vdots \\ x_{n,1} & x_{n,2} & \ldots & x_{n,m} \end{array}\right]⇝\left[x_{1,1},x_{1,2},\ldots ,x_{1,m},x_{2,1},x_{2,2},\ldots ,x_{2,m},\ldots ,x_{n,m}\right]$$

The overall firmware source code followed the standard life cycle for Arduino applications. Once the firmware was flashed to the microcontroller, we confirmed the correct workings using PlatformIO’s serial monitor feature to read the transmitted data.

### 2.4. Signal Preprocessing

This section first proposes a software architecture for the data processing pipeline and additional application logic, and then describes the rules for transforming the raw data stream into a usable data structure. It then explains the filtering process and conversion to a grayscale image.

#### 2.4.1. Software Architecture

**Figure 5:**
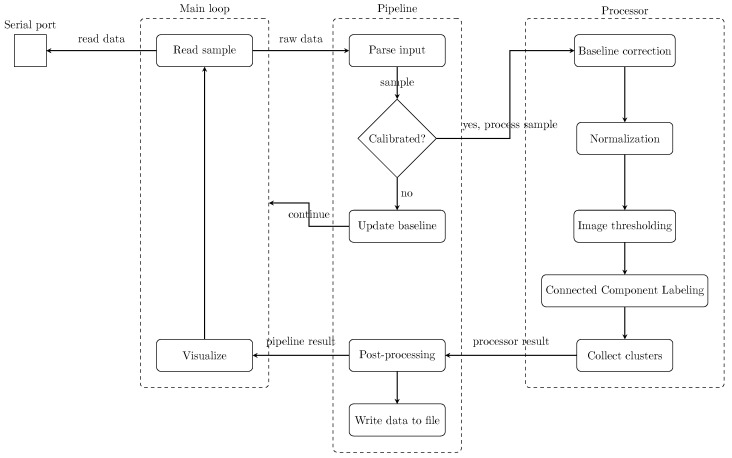
Conceptual model of the software architecture.

**Figure 6:**
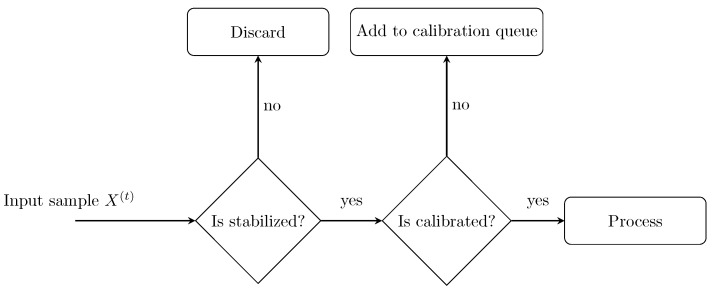
Flow diagram of a sample passing through the pipeline component.

The architecture was designed with modularity and maintainability in mind, ensuring that core functions such as reading, preprocessing, analysis, and rendering were logically separated and extensible. As shown in Figure 5, the application is structured into three main components: the main loop, the pipeline, and the processor. The main loop manages overall execution, including sensor communication, coordination of processing steps, and real-time visualization. The pipeline component is responsible for parsing raw sensor input and passing structured data forward and also contains logic for system initialization and calibration. The processor, acting as the core analytical unit, handles anomaly detection and clustering based on the processed data. The pipeline’s responsibility was to receive raw input data, parse it into the data structure described in Section 2.4.2, and pass it to the processor. Additional initialization logic was necessary to accommodate the collection of baseline samples described in Section 2.4.3. The initialization logic also included a mechanism to discard the first few samples to wait for stabilized values from the sensor. Figure 6 illustrates how a sample $X^{\left(t\right)}$ passes through the pipeline component. The first 5 samples were discarded as a precaution to unstable values from the sensor.

After the stabilization phase, the application switched to the calibration phase and collected the samples to calculate the baseline described in Section 2.4.3. After completing the calibration phase and computing the baseline, the processor component directly processed all subsequent samples. The stabilization and calibration phases required a mechanism to collect samples in a fixed-sized queue: adding new ones to the top and discarding the oldest ones. Python’s built-in collections module provides the deque that generalizes the stack and queue data structure. Its efficient and generalized implementation perfectly fits the purpose described above.

#### 2.4.2. Data Transformation

A sensor with *n* rows and *m* columns transmitted the sensor data as a row-wise concatenated list of comma-separated values. Let the n×m matrix $X\in R^{n\times m}$ represent the one-dimensional array, where the entry $x_{i,j}$ denotes the value of the sensor cell at row *i* and column *j*. The transformation process is depicted in Equation (3).

(3) $$\left[x_{1,1},x_{1,2},\ldots ,x_{1,m},x_{2,1},x_{2,2},\ldots ,x_{2,m},\ldots ,x_{n,m}\right]⇝\left[\begin{array}{cccc} x_{1,1} & x_{1,2} & \ldots & x_{1,m}\\ x_{2,1} & x_{2,2} & \ldots & x_{2,m}\\ \vdots & \vdots & \ddots & \vdots \\ x_{n,1} & x_{n,2} & \ldots & x_{n,m} \end{array}\right]$$

The raw input from the sensor arrives as a continuous stream of numerical values, which must be transformed into a structured format suitable for analysis. On the left-hand side of the data stream are the raw, comma-separated capacitance values received from the microcontroller, while the right-hand side shows the data matrix. For example, a sensor with a grid of size 12×21 means that the data matrix *X* has 252 entries. The microcontroller board sends data approximately every 16 ms and treats each sensor cell value as a 32 bit integer. Collecting the data stream over 60 s results in 945,000 values, implying a storage requirement of approximately 3.78 MB.

**Figure 7:**
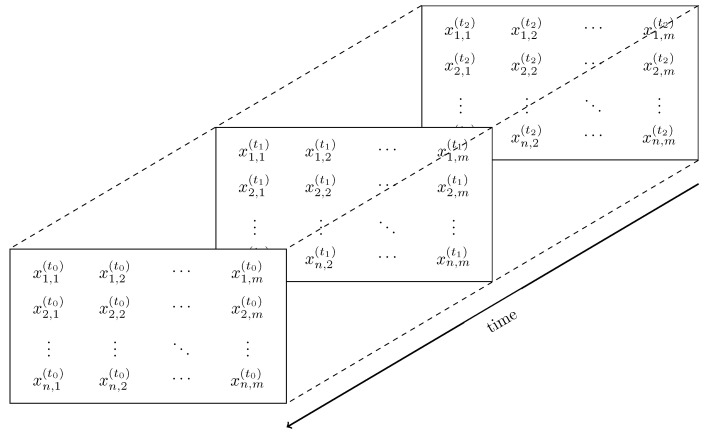
Sensor data stream observed from the time domain. The labels “$\left(t_{k}\right)$” of the samples $x_{i,j}$ indicate progressing time, whereas a higher numerical index *k* indicates a longer time in the past.

Figure 7 illustrates the data stream in the time domain and introduces the notation for indexing samples over time: A sample *X* at time *t* is referred to as $X^{\left(t\right)}$, with the corresponding entries $x_{i,j}^{\left(t\right)}$. To indicate the sample’s age, the moment in time *t* is indexed with an age parameter $k\in N_{0}$ as $t_{k}$: $t_{0}$ denotes the most recent (latest) sample, while a higher index *k* corresponds to an older sample from further in the past. The established data structure and time-indexing notation simplify the formulations throughout this work.

#### 2.4.3. Signal Filtering

Sensor data collected from the soft elastomeric array are inherently noisy due to external interferences and inconsistencies introduced during fabrication. Variations in the amount and shape of conductive material across sensing nodes lead to nonuniform signal behavior, making preprocessing essential before further analysis.

Signal filtering methods are generally categorized into probabilistic and analytical approaches. Probabilistic filters, such as the Wiener and Kalman filters, rely on statistical models and require prior knowledge of the system’s dynamics [35]. Analytical filters, including the Savitzky–Golay and Bessel filters, use numerical approximations to smooth the signal [36,37].

These classical approaches, however, pose limitations for real-time applications. Probabilistic methods assume known system behavior, while analytical methods often depend on window functions that reduce response time and compromise real-time performance. Since the sensor is still under development and lacks prior characterization, an empirical Impulse Response Function (IRF) was recorded by applying a short force to evaluate system behavior. Due to the lack of established sensor models and the need to maintain responsiveness, filters with large window sizes or recursive processing were avoided. A threshold-based filtering technique was adopted instead, offering a lightweight solution that suppresses low-level fluctuations while preserving relevant signal features for localization.

Thus, the solution to the signal filtering was to define a calibration phase to capture the sensor’s behavior under the expected environmental conditions at rest. The calibration phase collected the samples $X^{\left(1\right)},\ldots ,X^{\left(T\right)}$, where $X^{\left(t\right)}\in R^{n\times m},\forall t\in 1,\ldots ,T$. Based on these calibration samples, the sample mean and sample variance were computed for every sensor cell (i,j),∀i=1,…,n,j=1,…,m, as follows:

(4) $$\begin{array}{cc} {\bar{\mu }}_{i,j} & =\frac{1}{N}\sum_{t=1}^{T}x_{i,j}^{\left(t\right)}, \end{array}$$

(5) $$\begin{array}{cc} {\bar{\sigma }}_{i,j}^{2} & =\frac{1}{N}\sum_{t=1}^{T}{\left(x_{i,j}^{\left(t\right)}-{\bar{\mu }}_{i,j}\right)}^{2}. \end{array}$$

Equations (4) and (5) formalize the computation of the baseline. Assuming that the sensor’s environment remained stable during the calibration phase and that the noise followed a normal distribution, the following threshold filter was constructed:

(6) $$f_{\mathrm{threshold}}\left(x_{i,j}\right)=\left\{\begin{array}{cc} 0, & \;x_{i,j}\leq {\bar{\mu }}_{i,j}+c\;\cdot \;{\bar{\sigma }}_{i,j}^{2}\\ x_{i,j}, & \;\mathrm{otherwise} \end{array}\right.$$

where c∈R determines the level of sensitivity against noise. A higher value for *c* translates to a higher threshold that the signal must overcome not to be grounded (set to 0). The threshold function was explicitly designed to have a one-sided cut-off because higher sensor readings suggest the presence of contact points.

**Figure 8:**
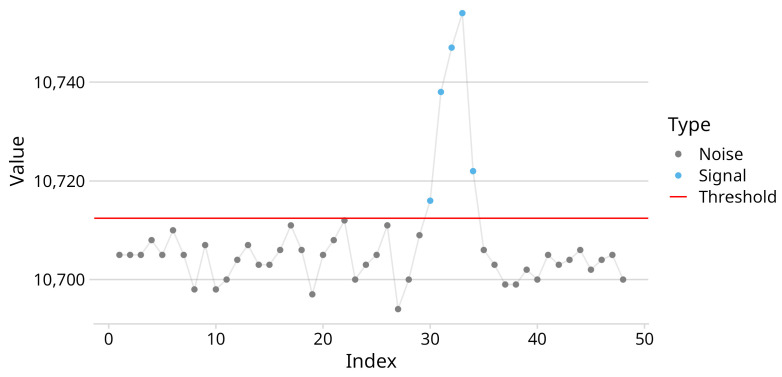
Threshold filter applied on input data $x_{i,j}^{\left(t\right)}$. Observations below the threshold are classified as noise.

After the calibration phase, all future samples passed through the threshold function defined in Equation (6). Figure 8 visualizes the result of applying the threshold filter to noisy data. This approach was fast and did not compromise the signal when actual force was applied, but it falls short when requiring high sensitivity. The next step after filtration was the segregation of signal from noise. The chosen approach was to represent the sample $X^{\left(t\right)}$ as a grayscale image and apply computer vision algorithms for anomaly detection. The filtered sample $X^{\left(t\right)}$ was then scaled to [0,1] with a min–max scaler and multiplied by 255. Because the data that fell below the threshold were set to 0, this value was exclusively reserved for noise and unchanged by the min–max scaler and multiplication.

### 2.5. Force Point Detection and Localization

The preceding steps in the processing pipeline result in a normalized grayscale image, where the intensity of each pixel reflects the relative capacitance change in the corresponding sensor cell. This image is further analyzed using computer vision techniques to identify areas where force is applied. These methods enable the segmentation, localization, and grouping of activated sensor regions representing surface pressure points. The goal is to transform the continuous image data into discrete, analyzable contact points that can be tracked over time. This section presents the applied techniques for anomaly detection, region clustering, and tracking in detail.

#### 2.5.1. Otsu’s Method and Connected Component Labeling

One of the problem statements in image processing is converting grayscale images to binary format while expressing as much detail from the original image as possible. The goal is to find an optimal threshold to separate the grayscale pixel values into black and white. Otsu’s method finds the best threshold by sequentially searching for a threshold value that maximizes the between-class variance of pixel values [38]. The maximization effectively separates pixels into foreground and background regions in a grayscale image.

**Figure 9:**
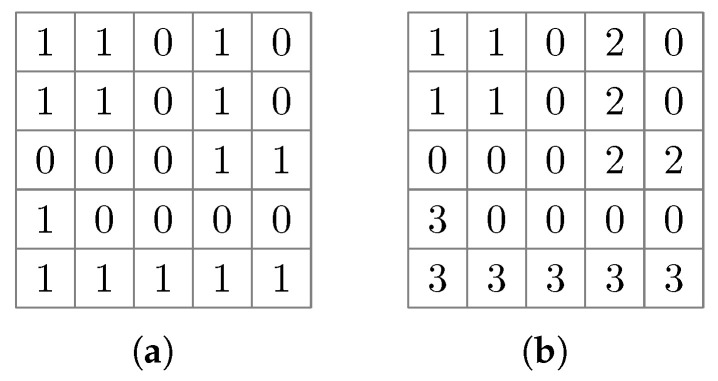
Illustration of applying the connected components labeling algorithm to a symbolic binary image (**a**); (**b**) is the output of the connected components operator.

Given a binary image with white pixels representing the foreground and black pixels representing the background, the Connected Component Labeling (CCL) algorithm groups the pixels into regions [39]. Figure 9 illustrates the grouping of connected foreground pixels labeled “1”. Each region has a distinct label, and adjacent pixels of a region have the same label.

**Figure 10:**
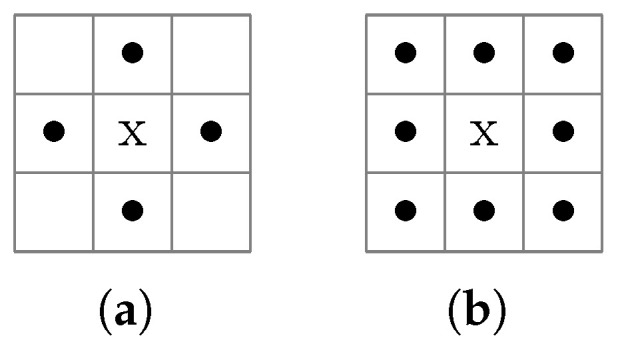
The central pixel *x* is connected to (**a**) 4 and (**b**) 8 neighboring pixels (•).

The algorithm takes two sequential passes over the grayscale input image. The first pass assigns new labels to the first pixel of each component and propagates labels from left to right and top to bottom according to the selected adjacency mode: when only considering the pixel neighbors of the four cardinal directions, then we speak of 4-connectedness. If additional diagonal directions are considered, it is called 8-connectedness. Figure 10 illustrates the concepts of 4- and 8-connectedness. Due to its sequential nature, a connected region may contain parts with different labels. The second pass sets up equivalency tables of labels to uniquely identify the connected components. The classical CCL algorithm described above requires two passes over the binary input image. Thus, for an n×n image, the algorithm has a $O(n^{2})$ complexity. The scikit-image Python package and similar software implementations utilize CCL algorithms optimized for time or space efficiency [40,41].

#### 2.5.2. Cluster Tracking

**Figure 11:**
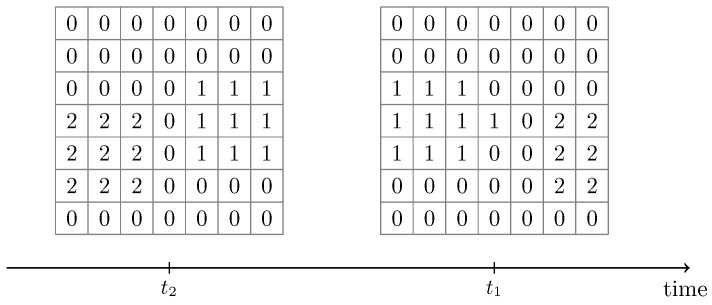
Tracking moving clusters from the CCL output at time $t_{2}$ and $t_{1}$. Background pixels are marked with “0”, pixels from contact points are marked with “1” or “2” and belong to cluster $C_{1}$ and $C_{2}$, respectively. The change of labels from time $t_{2}$ to $t_{1}$ is due to the sequential nature of the CCL algorithm.

The CCL algorithm performs sequential scans of the input image, and even slight variations in the input data may lead to different cluster label assignments (see Figure 11). The unstable label assignment posed a challenge for collecting cluster statistics over time.

Performing CCL for a sample $X^{\left(t\right)}$ resulted in pixel clusters $C_{i}$, where i∈{0,…,k} denotes the cluster label. The clusters are disjoint, and all pixels in a cluster have the same label. Only clusters holding signal pixels were of interest. Thus, the following analysis omits the background clusters $C_{0}$. The time-indexing notation from the samples is extended to the clusters: a cluster $C_{i}$ obtained from $X^{\left(t\right)}$ is referred to as $C_{i}^{\left(t\right)}$. Figure 11 illustrates the membership assignment at different points in time with moving clusters. One would expect the clusters to retain their labels even if they move over time, but the CCL algorithm does not guarantee this.

The task of the tracking algorithm was to match the previously recorded clusters $C_{i}^{\left(t_{2}\right)}$ with the current clusters $C_{j}^{\left(t_{1}\right)}$ for all i,j∈{1,…,k}. Utilization of the Jaccard similarity score, also known as the Jaccard index or Jaccard score, solved the problem of matching previous clusters to current ones [42]. The Jaccard score is defined by

(7) $$J\left(C_{i}^{\left(t\right)},C_{j}^{\left(t\right)}\right)=\frac{\left|C_{i}^{\left(t\right)}\cap C_{j}^{\left(t\right)}\right|}{\left|C_{i}^{\left(t\right)}\cup C_{j}^{\left(t\right)}\right|},\;\forall i,j\in \left\{1,\ldots ,k\right\}$$

and depicts the ratio of shared pixels to the total number of pixels of $C_{i}^{\left(t\right)}$ and $C_{j}^{\left(t\right)}$.

Table 1 shows the calculated Jaccard scores between the clusters depicted in Figure 11. The row labels contain the past clusters, while placing the last known clusters in the columns. Finding the best-matching past cluster for, e.g., $C_{1}^{\left(t_{1}\right)}$, is only a matter of identifying the maximum value in the corresponding column. The cluster from the corresponding row, in this case, $C_{2}^{\left(t_{2}\right)}$, is the most similar to $C_{1}^{\left(t_{1}\right)}$. New clusters with no matching cluster were sequentially assigned a new label, starting at the highest cluster label from time $t_{1}$.

A possible issue of this cluster-tracking approach is the splitting of cluster $C_{i}^{\left(t_{2}\right)}$ into $C_{i_{1}}^{\left(t_{1}\right)}$ and $C_{i_{2}}^{\left(t_{1}\right)}$ and vice versa.

Algorithm 1 was devised to prevent labeling inconsistencies when dealing with moving clusters. However, implementation details of the methods were omitted to make the information more readable. The findNewLabel function assigns a label to each cluster based on the most similar existing cluster $c_{\mathrm{similar}}$ and previously used labels; it either reuses the label of $c_{\mathrm{similar}}$ or generates a new one in sequential order. The variable label represents the assigned label of a given cluster. Additionally, the sortLabels method organizes the clusters in $C^{\left(t_{1}\right)}$ in descending order based on their labels.

Suppose $C_{i}^{\left(t_{2}\right)}$ splits into two new clusters, $C_{i_{1}}^{\left(t_{1}\right)}$ and $C_{i_{2}}^{\left(t_{1}\right)}$, with $C_{i_{1}}^{\left(t_{1}\right)}\cap C_{i_{2}}^{\left(t_{1}\right)}=\emptyset$ and $C_{i_{1}}^{\left(t_{1}\right)}\cap C_{i}^{\left(t_{2}\right)}\neq \emptyset ,C_{i_{2}}^{\left(t_{1}\right)}\cap C_{i}^{\left(t_{2}\right)}\neq \emptyset$. Both clusters have the highest similarity to their parent cluster and would inherit the cluster label from their predecessor—an inconsistency. This case was avoided by tracking the assigned labels and adequately implementing the findNewLabel function.

Although various unsupervised learning algorithms such as DBSCAN, hierarchical clustering, and k-means are commonly used for anomaly detection and signal segmentation [43,44,45], they were not employed in this work. These methods require careful tuning of hyperparameters, such as the number of clusters or the neighborhood radius, which can vary significantly across different datasets. Moreover, the computational cost of parameter estimation and model execution is typically not well suited for real-time environments. For these reasons, they were excluded from further consideration in favor of more lightweight and responsive techniques.
**Algorithm 1:** Cluster tracking algorithm
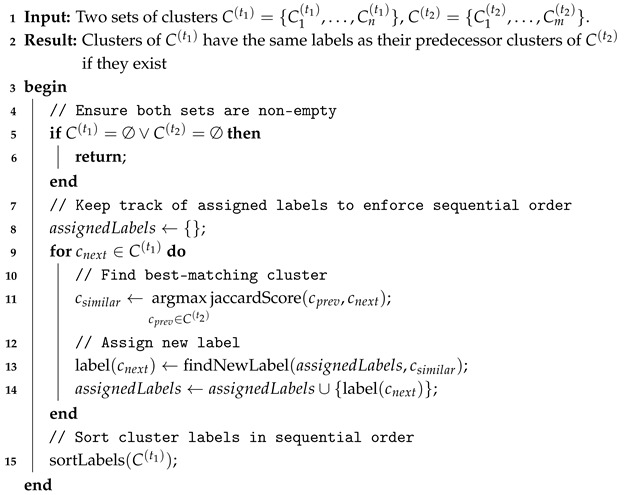


### 2.6. Machine Learning Models for Correlation Factor

This employs a data-driven approach to characterize force using machine learning models applied to capacitive sensor data. The methodology is structured into three primary stages: data preprocessing, model training, and performance evaluation. The known normal force is applied using the UR3e and the AXIA 80 force sensor [46]. The Ratio of Mutual Capacitance (RMC) and force data are recorded for different sensor nodes. A moving average filtering technique is applied to refine the dataset. This approach smooths high-frequency fluctuations and minimizes random noise, enhancing signal stability and ensuring that the extracted force–capacitance relationship remains consistent.

Once the data are preprocessed, supervised learning models are trained to establish a mathematical relationship between the capacitance readings and the applied force. The models evaluated in this study include Linear Regression, Support Vector Machines (SVM), Decision Tree Regression, and Gaussian Process Regression (GPR) [47]. Linear Regression is widely used for modeling proportional dependencies between variables and is commonly applied in sensor calibration scenarios [48]. However, due to the nonlinearity in the capacitance–force relationship, more advanced models such as SVM with a linear kernel, Decision Tree Regression, and GPR are considered. SVM is effective for high-dimensional regression problems, while Decision Trees are known for capturing complex, nonlinear dependencies. GPR, being a probabilistic approach, is particularly advantageous when dealing with noisy datasets, as it provides uncertainty estimates and predictions [49].

The models are assessed using key statistical metrics, including Root Mean Squared Error (RMSE), coefficient of determination ($R^{2}$), and slope estimation. RMSE quantifies the deviation between predicted and actual force values, indicating overall model accuracy. $R^{2}$ evaluates how well the model explains the variance in the capacitance–force relationship. The slope estimation is a crucial correlation factor, allowing capacitance measurements to be translated into force values. This ensures that the developed model can be effectively integrated into sensor-based applications for force estimation. This study compares different regression models through this structured methodology, identifying the most suitable approach for force estimation from capacitance-based sensor readings. The insights gained from this analysis contribute to developing more precise and reliable force-sensing systems, enhancing their applicability in real-world tactile sensing and robotics applications.

## 3. Results

### 3.1. Data Processing

**Figure 12:**
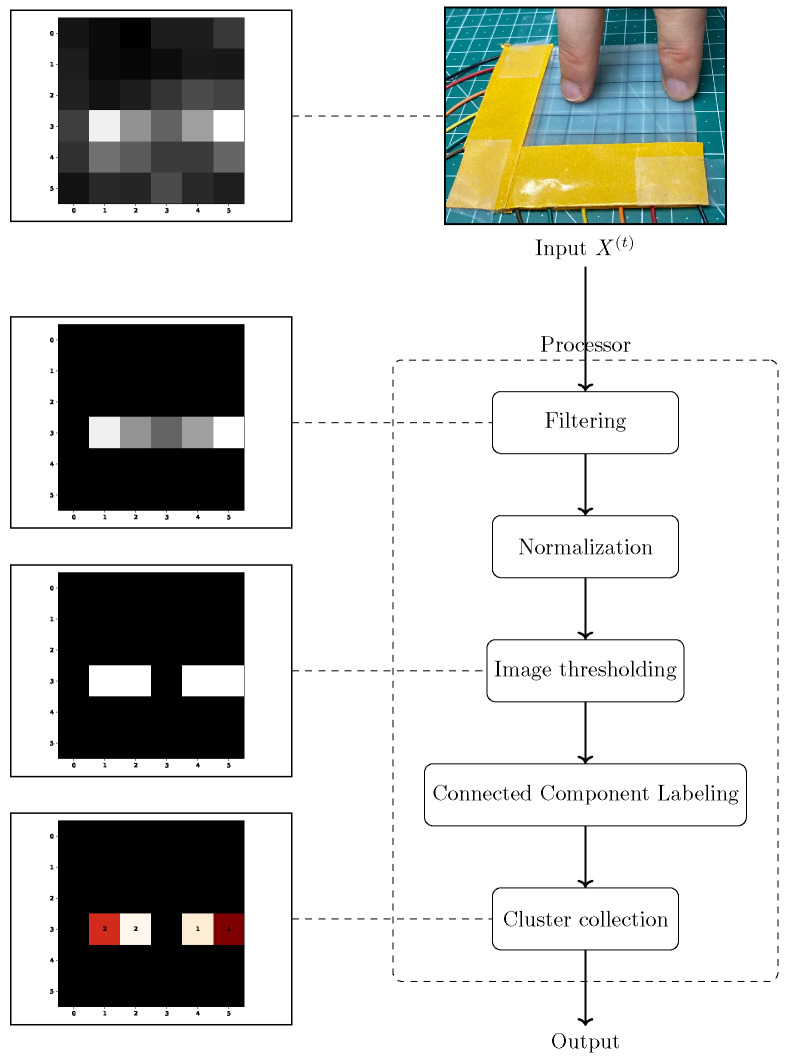
Overview of the processing pipeline for an input sample $X^{\left(t\right)}$; top images show the interaction generating input, and left images illustrate each processing stage. The greyscale heatmaps represent intermediate stages of the processing pipeline, while the final stage uses a color map to represent pressure intensity, with warmer colors indicating higher pressure areas.

The processor is the core component of the Python application that performs the anomaly detection and clustering on the input data. The core functions used in the stages reflect the algorithms discussed in Section 2.4.3 and Section 2.5; Figure 12 illustrates its relationship.

The pipeline component provided the baseline measurements for the processor component. Thus, the processor only had to calculate the baseline described in Section 2.4.3. Incoming samples $X^{\left(t\right)}$ were treated by applying the baseline correction via the threshold filter introduced in Equation (6). The next step normalized the entries of $X^{\left(t\right)}$ into the range [0,1], then converted the data to a grayscale image. Further differentiation of the signal from noise was achieved by applying Otsu’s image thresholding algorithm (refer to Section 2.5.1) on the grayscale image. The output of this stage was a binary image, with black pixels representing background noise (label “0”) and white pixels representing the signal (label “1”). The individual pixels were then summarized into groups with the CCL algorithm that required a binary input format. Consistent cluster labeling enabled the collection of additional statistics about the clusters, such as average cell load or cluster size.

### 3.2. Data Visualization

**Figure 13:**
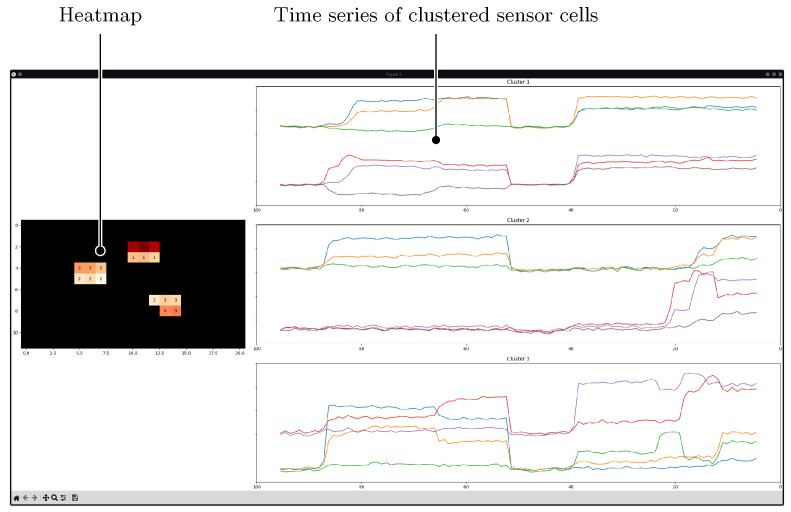
Python application for visualizing sensor cell responses over time. The heatmap on the left presents real-time pressure distribution, where warmer colors indicate higher pressure. The three time series plots on the right display sensor readings for the first three clusters, with each time series shown in a unique color to distinguish individual sensor cells.

One of the research questions focused on finding an appropriate way to visualize the sensor data. A heatmap was selected to show the pressure distribution because it is easy to interpret and reflects the current state of the data. Furthermore, time series graphs were shown next to the heatmap to illustrate the changes in individual sensor cells over time. Figure 13 shows the combined visualization methods that enabled pressure distribution analysis both through a snapshot (the heatmap) and through observation of the overall behavior over time.

To ensure smooth real-time performance, the visualization was optimized by limiting the time series display to three panels, each showing sensor data lines grouped by cluster. The heatmap legend and y-axis labels were omitted to maintain high rendering speed during animation using matplotlib. The data passed through a threshold filter (Equation (6)); the values were positive, and the pressure was proportional to the sensor readings. A heatmap with a sequential color scale effectively communicated continuous pressure differences between several locations [50]. The sequential color scale had varying hues of red that optimally mapped the two border states: low pressure (light) and high pressure (dark).

**Figure 14:**
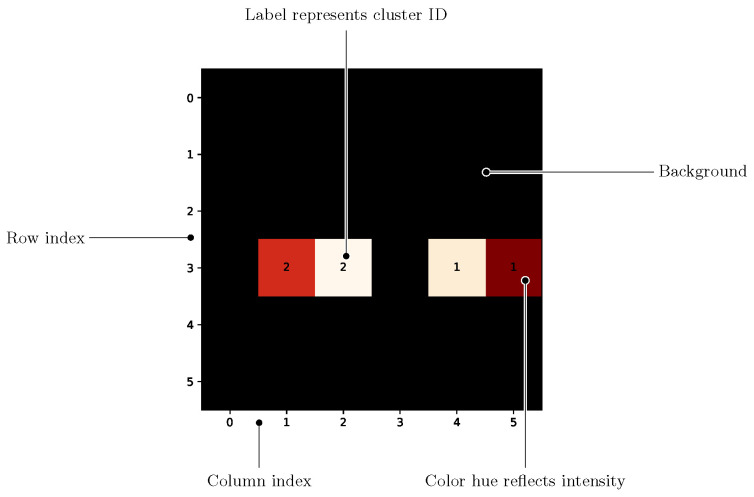
Heatmap with annotations highlighting the relevant components. Warmer colors indicate areas of higher pressure.

There were some tweaks that differentiated the visualization from a usual heatmap: The clustered anomalies were indicated via labels in the cells of the heatmap; they also provide a means to connect the clusters from the heatmap with the time series graphs of the individual sensor cell values. Data points not classified as anomalies, the background noise, were displayed as black pixels. Figure 14 shows the discussed approach with an example.

### 3.3. Algorithm Performance

**Figure 15:**
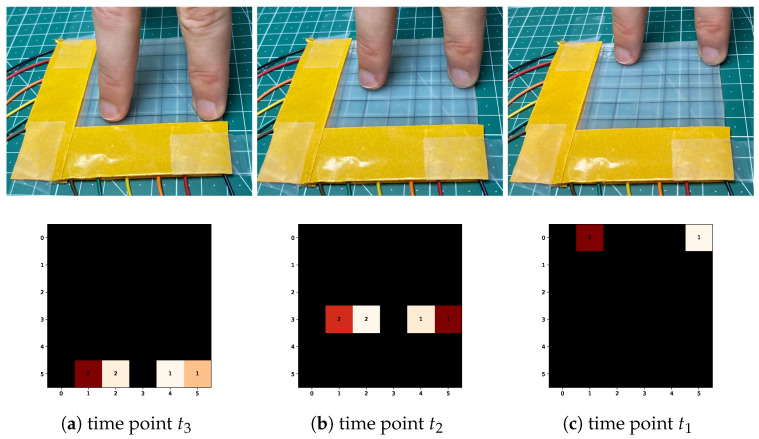
Snapshots of two fingers gliding over the MUCA touchpad (**first row**) with the respective anomaly clusters (**second row**) at time points $t_{3},t_{2}$, and $t_{1}$. Warmer colors in the heatmaps indicate areas of higher pressure.

The performance of the processing pipeline was evaluated to verify that the application and the sensor can be operated in real time. The application’s real-time capabilities were evaluated in a field test by applying force to one or more punctual locations moving across the sensor. Figure 15 illustrates snapshots of such a test with the input gesture and the corresponding output. Low latency between input gestures and the visualizations conveyed a smooth feedback loop. A shortcoming of the application was that abrupt changes to the input, e.g., rapidly enlarging the pressure area or quickly moving from one point to another, caused flickering in the visualization, which was challenging to interpret.

**Figure 16:**
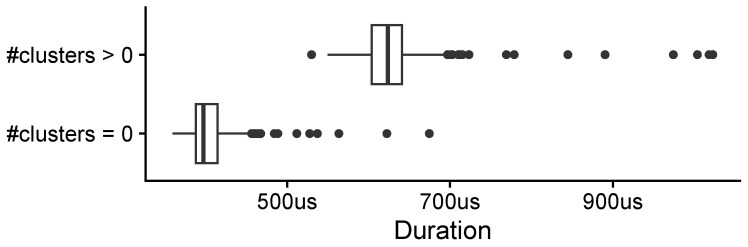
Processing time comparison for clustered and non-clustered samples.

The processing speed of the processor component was evaluated using data from the soft sensor prototype shown in Figure 3. Python’s built-in time.perf_counter_ns method was used to measure the algorithm’s time performance, which returns the current clock value with the highest possible resolution of nanoseconds. The duration of processing a sample $X^{\left(t\right)}$ corresponds to the duration it took the processor component to process and cluster the data. Figure 16 compares the pipeline’s processing duration for different input types. Samples without any detected clusters (n=437) required an average processing time of approximately 404.7 μs, with a standard deviation of 29.9 μs. In contrast, samples containing contact point clusters (n=220) typically involving 5±3 clusters took an average of 627.2 μs, with a standard deviation of 46.4 μs. These results demonstrate that processing time increases with the complexity of the sensor input yet remains well below 1 ms, ensuring compatibility with real-time applications.

Interactions (contact points) with the soft sensor caused anomalies in the data, and thus, the application performed the cluster analysis and tracking. Figure 16 compares the processing speed of the pipeline when it received samples with and without clusters (which we synonymously use for contact points). When no anomalies were present, the pipeline skipped the clustering step and achieved faster processing speed. In comparison, the clustering and post-processing of samples containing contact points required more time. Note that the processor’s performance depends on the sensor resolution and, if present, the number of clusters.

### 3.4. Tactile Sensor Performance Evaluation

A 5 mm-diameter probe, 3D-printed using Prusament PLA Galaxy Silver, was used to evaluate the sensor for force application. The probe tip featured a 1 mm fillet to ensure smooth contact without sharp edges. The material was non-conductive and moderately stiff, and environmental electrostatic effects were minimized during controlled testing conditions without additional grounding. While force characterization was performed under controlled laboratory conditions minimizing electrostatic effects, environmental factors inherently influence capacitive sensing. The tactile sensor array comprises 16 individual sensors arranged in a structured grid format, though for conciseness, only the results from four representative sensors are presented in this study. The observed trends from these selected sensors are consistent across the entire array, indicating uniform performance across all sensing nodes.

**Figure 17:**
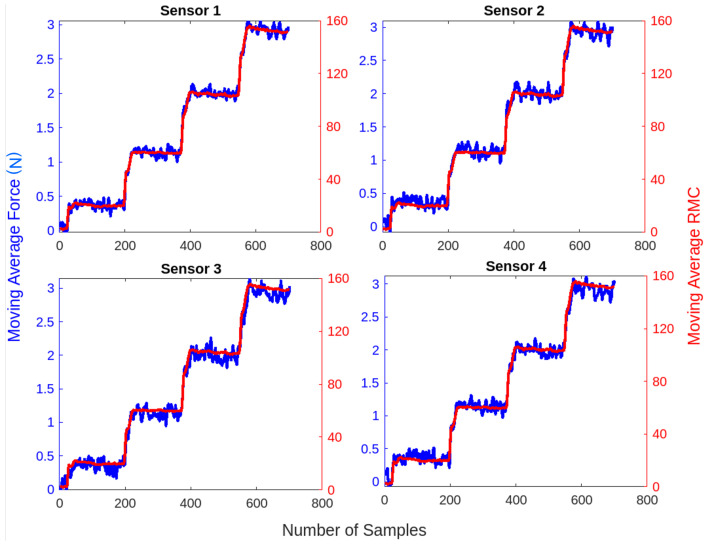
Force and RMC response of tactile sensors over sampled data.

The experimental setup employed a robotic arm to apply normal forces ranging from 0.1 N to 3 N, while the corresponding RMC exhibited a peak value of 150. The limit of detection was around 0.1 N. The moving average approach filters out noise and improves signal stability. Figure 17 presents the relationship between normal force and mutual capacitance ratio for four selected sensors. The consistency of the results across the different sensors suggests a high level of repeatability in the capacitive response to the applied force.

### 3.5. Force Characterization Using Machine Learning Models

Multiple machine learning regression models were evaluated to establish a reliable correlation between mutual capacitance and applied force. The selected models include Linear Regression, SVM, Decision Tree Regression, and GPR, as detailed in Section 2.6. Each model was assessed based on key performance metrics: RMSE, $R^{2}$, and correlation factor, which enables force estimation from capacitance measurements. Linear Regression and SVM provided predictable results, assuming a linear relationship between capacitance and force. Regression models were trained on 70% of the collected data and evaluated on the remaining 30% testing set. Repeatability was assessed by repeating force applications 100 times at the same force levels, ensuring consistent sensor responses. Future work will include detailed hysteresis characterization and broader validation under dynamic loading conditions. Their regression lines were relatively smooth and consistent, with $R^{2}$ values exceeding 0.995 across all tested sensors, indicating strong predictive power. However, these models exhibited limitations in capturing potential nonlinearity in sensor responses. In contrast, Decision Tree Regression and GPR demonstrated fluctuating trends in predicted force values, resulting in irregular patterns. This behavior arises due to the nature of these models; Decision Tree Regression splits the data into discrete decision boundaries, causing step-like transitions, whereas GPR is a probabilistic model that estimates function fits, often leading to sensitivity to noise and localized oscillations.

**Figure 18:**
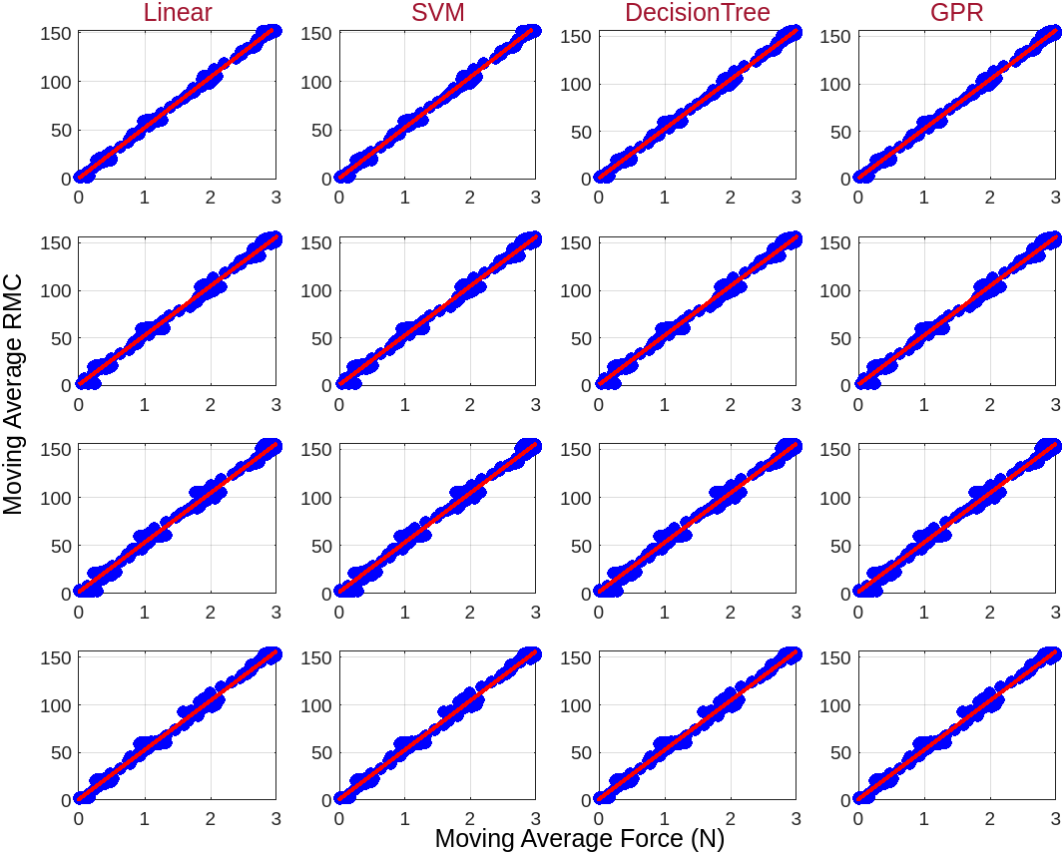
Comparison of machine learning models for force and RMC relationship prediction. Blue dots represent actual data, and red lines indicate model predictions.

The close performance of different machine learning models in Figure 18 reflects the fabricated sensors’ strong linear and repeatable behavior, as seen in the experimental data. Benchmarking multiple models ensures validation of the sensor’s robustness and assesses the potential for capturing any minor nonlinearities. Although all models achieved $R^{2}$ values above 0.99, Decision Tree Regression balanced accuracy and deployment suitability for embedded real-time applications. The results summarized in Table 2 indicate that Decision Tree Regression achieved the lowest RMSE values, ranging between 0.0446 and 0.0585, confirming minimal prediction error. Its average $R^{2}$ value of 0.9996 also suggests a strong ability to explain variance in the force–capacitance relationship. As a result, Decision Tree Regression was selected as the most effective model for force estimation, balancing accuracy, and predictive stability. Unlike purely linear models, it captures subtle variations while maintaining high reliability. The average slope obtained from the Decision Tree model was 51.76, which serves as the correlation factor for translating capacitance measurements into force values.

## 4. Conclusions

This study presents a robust real-time system for detecting and localizing pressure points using a soft elastomeric capacitive sensor array developed to address the limitations of existing prosthetic and tactile sensing technologies. The sensor, fabricated using Dragon Skin and copper yarn electrodes, demonstrated consistent force sensitivity across a 6×6 matrix with a measurable range from 0.1 to 3 N. Data acquisition through a custom hardware setup and Arduino-based microcontroller enabled continuous transmission of raw mutual capacitance data, which were processed using a lightweight Python pipeline. The signal preprocessing approach incorporated threshold-based filtering and image conversion techniques, followed by Otsu’s binarization and a Connected Component Labeling algorithm. The system maintained an average processing time of approximately 627 μs for clustered input samples, ensuring responsiveness under dynamic conditions and within the real-time threshold of 1 ms. A cluster-tracking algorithm was implemented to provide consistent labeling over time using Jaccard similarity, effectively managing pressure point position and intensity variations. Machine learning models were employed to correlate sensor output with applied force for force estimation. Among the tested models, Decision Tree Regression outperformed others, achieving an $R^{2}$ of 0.9996, making it suitable for embedded systems requiring low latency and interpretable outputs. Visualization tools, including heatmaps and time series plots, enabled intuitive interpretation of dynamic tactile inputs. The proposed system is scalable and compatible with curved and deformable surfaces, making it applicable to sensorized prosthetic liners, wearable health monitoring, and adaptive soft robotic grippers. The presented sensor demonstrates real-time detection and localization as an initial proof of concept in the 0.1–3 N range. The design was refined in subsequent work to extend the force sensing capability up to 15 N with improved mechanical robustness and EMI shielding [46]. Ongoing developments aim to further increase the range to 25 N, with integration directly into the liner of prosthetic limbs. Future work may focus on miniaturizing the electronics, extending the sensor grid resolution, and deploying the platform in real-world prosthetic fittings. This work marks a significant step toward intelligent tactile systems that enhance user comfort, interaction feedback, and safety in biomedical and robotic domains.

## Figures and Tables

**Table 1:** Jaccard scores for clusters shown in Figure 11. The entries represent pairwise Jaccard indices between clusters at two time steps: the row headers ($C_{i}^{\left(t_{2}\right)}$) denote clusters at time $t_{2}$, and the column headers ($C_{j}^{\left(t_{1}\right)}$) denote clusters at time $t_{1}$. The column-wise maximum indicates the best-matching cluster for each current-time component.

Connected Component	$C_{1}^{\left(t_{1}\right)}$	$C_{2}^{\left(t_{1}\right)}$
$C_{1}^{\left(t_{2}\right)}$	0.0	0.36
$C_{2}^{\left(t_{2}\right)}$	0.46	0.0

**Table 2:** Performance comparison of regression models for force estimation.

Sensor	Linear Regression ($R^{2}$, RMSE)	SVM ($R^{2}$, RMSE)	Decision Tree ($R^{2}$, RMSE)	GPR ($R^{2}$, RMSE)
Sensor 1	0.99769,0.1008	0.99769,0.1354	0.99969,0.0446	0.99944,0.0543
Sensor 2	0.99608,0.1344	0.99608,0.1493	0.99945,0.0559	0.9989,0.0744
Sensor 3	0.99474,0.1494	0.99474,0.1575	0.99951,0.0585	0.99883,0.0785
Sensor 4	0.99536,0.1292	0.99536,0.1478	0.9996,0.0482	0.999,0.0724

## Data Availability

The Python application was open-sourced under the MIT license on GitHub and is available at https://github.com/peterpf/capsensor-monitor (accessed on 20 March 2025) [51].

## Abbreviations

The following abbreviations are used in this manuscript:

CCL	Connected Component Labeling
DAQ	Data Acquisition System
GPR	Gaussian Process Regression
I^2^C	Inter-Integrated Circuit
RMC	Ratio of Mutual Capacitance
RMSE	Root Mean Squared Error
SVM	Support Vector Machines
